# Orphan peptide and G protein‐coupled receptor signalling in alcohol use disorder

**DOI:** 10.1111/bph.16301

**Published:** 2024-01-08

**Authors:** Roberta Goncalves Anversa, Xavier J. Maddern, Andrew J. Lawrence, Leigh C. Walker

**Affiliations:** ^1^ Florey Institute of Neuroscience and Mental Health Melbourne VIC Australia; ^2^ Florey Department of Neuroscience and Mental Health University of Melbourne Melbourne VIC Australia

**Keywords:** alcohol use disorder, CART, GPCR, GPR135, GPR158, GPR26, GPR6, GPR88, neuropeptide, orphan

## Abstract

Neuropeptides and G protein‐coupled receptors (GPCRs) have long been, and continue to be, one of the most popular target classes for drug discovery in CNS disorders, including alcohol use disorder (AUD). Yet, orphaned neuropeptide systems and receptors (oGPCR), which have no known cognate receptor or ligand, remain understudied in drug discovery and development. Orphan neuropeptides and oGPCRs are abundantly expressed within the brain and represent an unprecedented opportunity to address brain function and may hold potential as novel treatments for disease. Here, we describe the current literature regarding orphaned neuropeptides and oGPCRs implicated in AUD. Specifically, in this review, we focus on the orphaned neuropeptide cocaine‐ and amphetamine‐regulated transcript (CART), and several oGPCRs that have been directly implicated in AUD (GPR6, GPR26, GPR88, GPR139, GPR158) and discuss their potential and pitfalls as novel treatments, and progress in identifying their cognate receptors or ligands.

**Abbreviations**
AUD: alcohol use disorder
CART: cocaine‐ and amphetamine‐regulated transcript
CeA: central nucleus of the amygdala
MSN: medium spiny neuron
oGPCR: orphan GPCR
PFC: prefrontal cortex
SNpc: substantia nigra pars compacta
SNr: substantia nigra pars reticulata
VTA: ventral tegmental area

## 1 GPCR SIGNALLING IN ALCOHOL USE DISORDERS

Alcohol use disorders (AUDs) are characterised by the impulsivity and compulsivity to seek and consume alcohol despite negative consequences (Koob & Volkow, 2010). Globally, it is estimated that 107 million people have an AUD, accounting for 1.4% of the global population (Institute of Health Metrics and Evaluation [IHME], 2019). The socioeconomic impacts of alcohol use are vast; in 2019 alone approximately 2.4 million deaths globally were attributed to AUD (IHME, 2019), and the global economic burden is estimated at 2.6% of the gross domestic product annually (Manthey et al., 2021). Currently, there are three Food and Drug Administration (FDA)‐approved drugs for the treatment of AUD: disulfiram, acamprosate, and naltrexone. Disulfiram acts to inhibit aldehyde dehydrogenase, causing adverse reactions when alcohol is consumed (Jørgensen et al., 2011). Acamprosate has an unknown mechanism of action, although it does reduce craving in some individuals with AUD (Spanagel et al., 2014; Witkiewitz et al., 2012). Naltrexone acts to reduce alcohol craving and heavy drinking in some individuals with AUD predominantly through antagonism of the μ‐opioid receptor (Anton, 2008). Unfortunately, all these treatments suffer from limitations, including inadequate efficacy, adverse side effects, and low compliance, rendering them somewhat ineffective at a population level (Kranzler & Soyka, 2018; Walker & Lawrence, 2018). Further, the last compound approved by the FDA for AUD was acamprosate, almost 20 years ago, highlighting the lack of effective treatments progressing to approval (Witkiewitz et al., 2012).

GPCRs, also termed seven‐transmembrane (7TM) domain receptors, are the largest class of receptors in the mammalian genome (Alexander et al., 2019) and have long been of interest as pharmacological targets to treat neuropsychiatric disorders and other diseases. GPCRs are currently classed into five main categories based on phylogenetic studies, forming the GRAFS classification—Glutamate (Class C), Rhodopsin (Class A), Adhesion, Secretin, and Frizzled/Taste2 (Fredriksson et al., 2003). These dynamic receptors undergo conformational changes upon ligand binding, leading to downstream modulation of transducer proteins. These include the heterotrimeric G protein subunits α, β, and γ which, upon receptor activation, dissociate to α and βγ (Kolb et al., 2022). There are 16 distinct α subunits that are categorised into four families based on downstream signalling pathways: G_s_ (increases adenylyl cyclase activity and levels of cAMP), G_i/o_ (reduces adenylyl cyclase and cAMP), G_q_ (increases DAG and IP_3_ ), and G_12/13_ (activates Rho) (Kolb et al., 2022). As of 2017, there were 475 drugs that target GPCRs approved by the FDA, representing ~34% of all FDA‐approved drugs, acting on 108 unique GPCR targets (Hauser et al., 2017, 2018). These drugs target only a fraction of known GPCRs, with dopamine, serotonin (5‐HT), cannabinoid and opioid receptors being prominent targets for disorders of the brain, including AUD.

Currently, there are 412 trials listed on clinicaltrials.gov for treatment/assessment of alcohol use. Of these, 73 assess drug interventions and 44 (60%) target GPCR mechanisms including 5‐HT receptors (14/44, 31%), cannabinoid receptors (8/44, 18%), oxytocin receptors (6/44, 13%), and GLP1 receptor (4/44, 9%) (see Table 1 for summary). A variety of other GPCR targets for AUD are in preclinical development including, but not limited to, muscarinic receptors (Walker et al., 2020, 2023), neurotensin receptors (Rodriguez et al., 2023), and neurokinin receptors (Schank, 2020). These data highlight the potential of GPCR signalling to treat AUD, a topic that is being widely explored. However, one avenue of GPCR signalling that remains underexplored is the potential of orphan neuropeptides and GPCRs (oGPCRs) as novel targets for disease, including AUD.

**TABLE 1:** Compounds currently in clinical trial for alcohol use disorder.

NCT number	Compound	GPCR action	GPCR class	Receptor class	Study title
NCT03764098	Guanfacine	Yes	Class A	Adrenoceptors	Mechanistic Evaluation of Guanfacine on Drinking Behavior in Women and Men With Alcohol Use Disorders
NCT04827056	Dexmedetomidine	Yes	Class A	Adrenoceptors	Effect of Sublingual Formulation of Dexmedetomidine HCl (BXCL501) ‐ Alcohol Interaction Study
NCT04135846	Doxazosin	Yes	Class A	Adrenoceptors	Alpha‐1 Blockade for Alcohol Use Disorder (AUD)
NCT03137082	Guanfacine	Yes	Class A	Adrenoceptors	Guanfacine to Reduce Relapse Risk in Women With Alcohol Use Disorder (AUD)
NCT04793685	Prazosin	Yes	Class A	Adrenoceptors	Prazosin for Alcohol Use Disorder With Withdrawal Symptoms
NCT05317546	Cannabidiol	Yes	Class A	Cannabinoid	Cannabidiol in Youth Alcohol Use Disorder
NCT04873453	Cannabidiol	Yes	Class A	Cannabinoid	CBD for the Treatment of Alcohol Use Disorder
NCT05613608	Cannabidiol	Yes	Class A	Cannabinoid	Alcohol Use Disorder and Cannabidiol
NCT05159830	Cannabidiol	Yes	Class A	Cannabinoid	Cannabidiol for Reducing Drinking in Alcohol Use Disorder
NCT05387148	Cannabidiol	Yes	Class A	Cannabinoid	The Efficacy and Neurobehavioural Mechanism of Cannabidiol (CBD) for Alcohol Dependence
NCT05860699	Cannabidiol	Yes	Class A	Cannabinoid	Cannabidiol as an add‐on Treatment During Inpatient Alcohol Cessation in Patients With Severe Alcohol Use Disorder: a Phase ii Trial
NCT04603781	Cannabidiol	Yes	Class A	Cannabinoid	CBD Oil for Reducing Emotional Impact of COVID‐19
NCT05781009	Pregnenolone	Yes	Class A	Cannabiniod	Pregnenolone for the Treatment of Alcohol Use Disorder
NCT02461927	Naltrexone (+ ketamine)	Yes	Class A	Opioid	Ketamine for the Rapid Treatment of Major Depressive Disorder and Alcohol Use Disorder
NCT05919017	Naltrexone	Yes	Class A	Opioid	Exploring the PK of Different Doses of Naltrexone in Patients With AUD
NCT05028062	Naltrexone	Yes	Class A	Opioid	Naltrexone in AUD Reward Drinkers
NCT05656534	Suvorexant	Yes	Class A	Orexin	Orexin Receptor Antagonists as Modulators of Threat Sensitivity in Individuals With Alcohol Use Disorder
NCT05312008	Oxytocin	Yes	Class A	Oxytocin	Does Oxytocin Alter Tolerance to or Motivation for Alcohol
NCT03878316	Oxytocin	Yes	Class A	Oxytocin	Intranasal Oxytocin for the Treatment of Alcohol Use Disorder
NCT04071119	Oxytocin	Yes	Class A	Oxytocin	Alcohol and Cigarette Craving During Oxytocin Treatment
NCT04523922	Oxytocin	Yes	Class A	Oxytocin	Oxytocin to Enhance Integrated Treatment for AUD and PTSD
NCT03846505	Oxytocin	Yes	Class A	Oxytocin	Oxytocin to Enhance Alcohol Behavioral Couple Therapy (ABCT)
NCT05093296	Oxytocin	Yes	Class A	Oxytocin	Oxytocin and Naltrexone: Investigation of Combined Effects on Stress‐ and Alcohol Cue‐induced Craving in Alcohol Use Disorder
NCT05674929	BPL‐003	Yes	Class A	Serotonin	An Open‐Label, Single Dose Study in Patients With Alcohol Use Disorder
NCT05913752	CMND‐100	Yes	Class A	Serotonin	A First in Human Study of CMND‐100 in Healthy and Alcohol Use Disorder (AUD) Subjects
NCT05474989	LSD	Yes	Class A	Serotonin	LSD Treatment for Persons With Alcohol Use Disorder
NCT05943665	MDMA	Yes	Class A	Serotonin	MDMA for AUD/PTSD Comorbidity
NCT05709353	MDMA	Yes	Class A	Serotonin	MDMA‐assisted Prolonged Exposure Therapy for Comorbid Alcohol Use Disorder and Post‐traumatic Stress Disorder
NCT05416229	Psilocybin	Yes	Class A	Serotonin	Psilocybin‐assisted Therapy for Treatment of Alcohol Use Disorder
NCT04410913	Psilocybin	Yes	Class A	Serotonin	Pilot Trial of Visual Healing® in Psilocybin‐assisted Therapy for Alcohol Use Disorder
NCT05646303	Psilocybin	Yes	Class A	Serotonin	Psilocybin‐Assisted Psychotherapy in Adults With Alcohol Use Disorder (AUD)
NCT04141501	Psilocybin	Yes	Class A	Serotonin	Clinical and Mechanistic Effects of Psilocybin in Alcohol Addicted Patients
NCT04718792	Psilocybin	Yes	Class A	Serotonin	Psilocybin for Treatment of Alcohol Use Disorder: a Feasibility Study
NCT04620759	Psilocybin	Yes	Class A	Serotonin	Psilocybin Treatment of Major Depressive Disorder With Co‐occurring Alcohol Use Disorder
NCT05421065	Psilocybin	Yes	Class A	Serotonin	Psilocybin‐Assisted vs Ketamine‐Assisted Psychotherapy for Alcohol Use Disorder
NCT04066192	Brexpiprazole	Yes	Class A	Serotonin & Dopamine	Brexpiprazole in Alcohol Use Disorder
NCT03526354	Brexpiprazole	Yes	Class A	Serotonin & Dopamine	Brexpiprazole Study
NCT05520775	Semaglutide	Yes	Secretin	Glucagon	Semaglutide for Alcohol Use Disorder
NCT05892432	Semaglutide	Yes	Secretin	Glucagon	Clinical Trial of Rybelsus (Semaglutide) Among Adults With Alcohol Use Disorder (AUD)
NCT05891587	Semaglutide	Yes	Secretin	Glucagon	Semaglutide Therapy for Alcohol Reduction ‐ Tulsa
NCT05895643	Semaglutide	Yes	Secretin	Glucagon	Does Semaglutide Reduce Alcohol Intake in Patients With Alcohol Use Disorder and Comorbid Obesity?
NCT04679142	Baclofen	Yes	Class C	GABA	Baclocur Post‐Authorisation Safety Study in Real‐life Settings in France
NCT04831684	GET73	Yes	Class C	Glutamate	Novel mGluR5 Modulator Effects on Alcohol Drinking and MRI Outcomes
NCT04218357	Probenecid	Yes	Frizzled/Taste2	Taste	Probenecid as Medication for Alcohol Use Disorder

**FIGURE 1:**
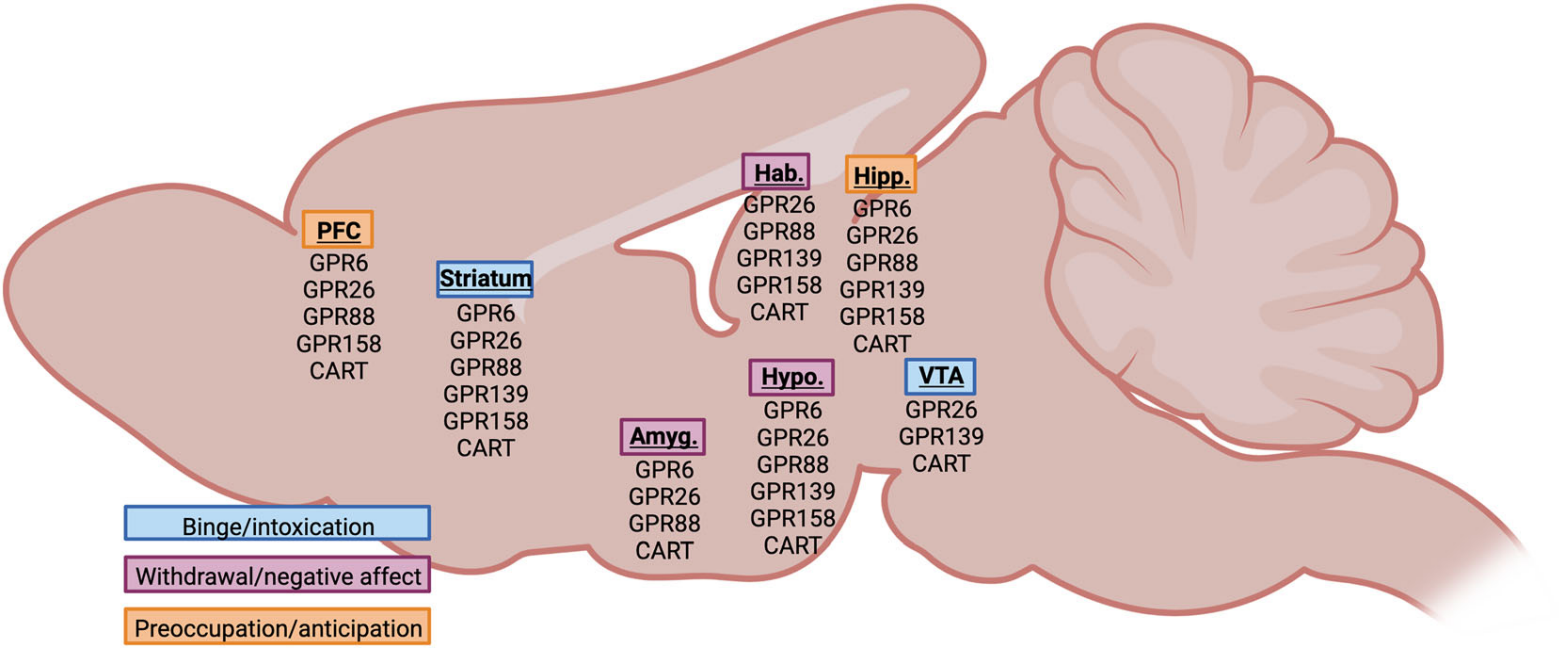
Orphan neuropeptide and receptor expression in key brain regions implicated in alcohol use disorder, colour‐coded according to the three key phases of the addiction cycle (binge/intoxication, withdrawal/negative affect, preoccupation/anticipation). Amyg., amygdala; CART, cocaine‐ and amphetamine‐regulated transcript; Hab., habenula; Hipp., hippocampus; Hypo., hypothalamus; PFC, prefrontal cortex; VTA, ventral tegmental area.

**FIGURE 2:**
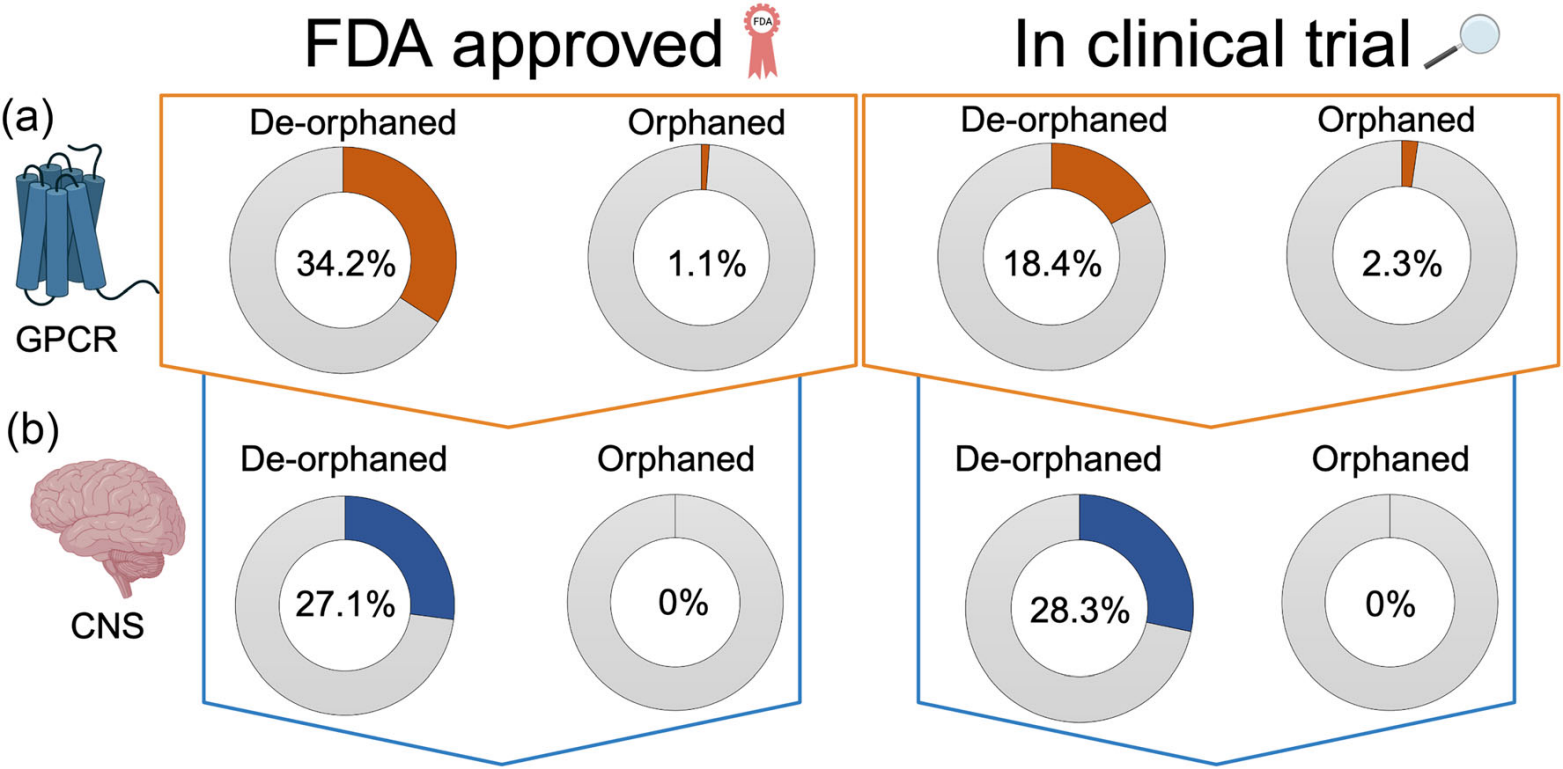
Food and Drug Administration (FDA)‐approved compounds and compounds in clinical trial targeting non‐olfactory G protein‐coupled receptors (GPCRs). (a) One hundred and six (34.2%) deorphaned GPCRs currently have an FDA‐approved compound to target them, compared with only one (1.1%) oGPCR (left). Further, there are currently 57 (18.4%) deorphaned GPCRs that have a drug in ongoing clinical trials, compared with only two (2.3%) drugs targeting oGPCR (right). (b) Of the total number of FDA‐approved drugs targeting deorphaned GPCRs, 130 (27.1%) are for CNS indications, while there are no approved drugs targeting oGPCRs for CNS indications. Further, 15 (28.3%) of drugs in clinical trials targeting deorphaned GPCRs are for CNS indications, while there are no drugs currently in ongoing clinical trials targeting oGPCRs for CNS indications.

Orphan neuropeptides are endogenous peptides that do not have a known receptor, while oGPCRs are receptors whose endogenous ligand is yet to be identified. Despite considerable efforts, more than 100 GPCRs remain orphaned, primarily within Rhodopsin (Class A) and Glutamate (Class C) GPCRs. The International Union of Basic and Clinical Pharmacology Committee on Receptor Nomenclature and Drug Classification (NC‐IUPHAR) consider an oGPCR de‐orphaned only when results are reproducible and criteria for likelihood of in vivo pairing are met (Alexander et al., 2019). Importantly, almost half of oGPCRs are expressed in the brain (Ehrlich et al., 2018; Hauser et al., 2017), including key regions associated with AUD (Figure 1), and these represent an unprecedented opportunity to address brain function and disease. Indeed, it is estimated that 57% of non‐olfactory GPCRs are still unexplored clinically, which includes a substantial proportion of orphan GPCRs that account for 22% of the total non‐olfactory GPCR population (Hauser et al., 2017). There are 106 deorphaned non‐olfactory GPCRs that currently have an FDA‐approved drug (34.2%), compared with only one oGPCR (1.1%) (Figure 2a). Further, 57 (18.4%) of deorphaned non‐olfactory GPCRs have a drug currently in clinical trial, compared with two oGPCRs (2.3%) (Figure 2a). Specifically for CNS indications, 130 (27.1%) of approved deorphaned non‐olfactory GPCR drugs are for CNS indications, but none target oGPCRs (Figure 2b). Of drugs currently in ongoing clinical trials targeting deorphaned non‐olfactory GPCRs, 15 (28.3%) of these drugs are for CNS indications, while there are no ongoing clinical trials for drugs targeting oGPCRs (Figure 2b). Here, we describe the current literature regarding orphaned neuropeptides and GPCRs implicated in AUD. Specifically, in this review, we focus on the orphaned neuropeptide cocaine‐and amphetamine‐regulated transcript (CART), and several oGPCRs that have been directly implicated in AUD ‐ GPR6, GPR26, GPR88, GPR139, GPR158 ‐ and discuss their potential and pitfalls as novel treatments.

## 2 ORPHANED NEUROPEPTIDES

### 2.1 Cocaine and amphetamine regulated transcript

The cocaine‐and‐amphetamine‐regulated‐transcript (CART) is a neuropeptide encoded by the *CARTPT* gene, which consists of three exons and two introns (Dominguez, 2006; Douglass & Daoud, 1996). Alternative splicing of this transcript results in two biologically active forms, CART_42–89_ and CART_49–89_ in humans, corresponding to CART_55–102_ and CART_62–102_ in rodents (Kuhar et al., 2002). Despite decades of research on CART since its initial isolation and sequencing in 1981 (Spiess et al., 1981), the cognate receptor(s) for CART remain disputed, and CART remains an orphaned ligand (Lau & Herzog, 2014; Ong & McNally, 2020). Although CART remains orphaned, it is suggested to signal via a G_i/o_‐coupled GPCR, linked to phosphorylation of the ERK pathway (Lakatos et al., 2005; Somalwar et al., 2018).

CART is densely expressed in reward‐related circuits critically involved in AUD, including the hypothalamus, nucleus accumbens, amygdala and Edinger–Westphal nucleus (Jaworski et al., 2008; Koylu et al., 1998; Millan & McNally, 2012; Walker et al., 2021). CART has also been heavily implicated in a range of drug‐related behaviours, including AUD (Kuhar, 2016; Ong & McNally, 2020; Vicentic & Jones, 2007). Early studies identified an association between an intron 1 polymorphism of the *CARTPT* gene and alcoholism in a Korean population (Jung et al., 2004). While tools to probe the function of CART are limited, several transgenic mouse lines have provided some insights to its function. Using two different transgenic *Cartpt* knockout (KO) mouse lines, *Cartpt* KO mice show reduced alcohol intake and preference in a two‐bottle choice procedure (Maddern et al., 2023; Salinas et al., 2014). Interestingly, sex differences arise in restricted binge alcohol access, with male *Cartpt* KO mice showing increased but female *Cartpt* KO mice decreased, alcohol intake, driven by bitter taste sensitivity in *Cartpt* KO female mice, involving CART signalling in the central nucleus of the amygdala (CeA; Maddern et al., 2023). CART is also implicated in alcohol seeking behaviours. Central infusions of CART peptide (CART_55–102_) reduced context‐induced reinstatement of alcohol seeking (King et al., 2010), whereas neutralisation of CART signalling (anti‐CART_55–102_) in the CeA reduced stress‐induced reinstatement of alcohol seeking in rats (Walker et al., 2021). This is likely to be linked to the role of CeA CART in alcohol withdrawal‐induced anxiety, as CeA CART_55–102_ neutralisation in the same species reduced social anxiety induced by alcohol withdrawal (Dandekar et al., 2008). Further, CART‐containing neurons in the arcuate nucleus of rats are activated following re‐exposure to stimuli previously associated with alcohol availability (Dayas et al., 2008). Together, these data highlight a role of CART in critical aspects for AUD from taste, consumption, alcohol‐induced withdrawal, and relapse. Despite the considerable number of publications implicating CART in a range of alcohol‐related behaviours, the exact neurobiological mechanism(s) mediating CART function remain unclear and are severely hindered by the lack of known cognate receptor(s) (Ong & McNally, 2020).

Recently, two orphaned GPCRs, GPR68 (Foster et al., 2019) and GPR160 (Yosten et al., 2020), have been proposed as putative CART receptors. GPR68 is a ubiquitously expressed proton‐sensitive receptor in brain neurons (Wang et al., 2020) and is expressed in key regions where CART terminals are located, including the striatum, amygdala and hippocampus. Additionally, GPR68 holds many characteristics of a peptide‐activated GPCR (Foster et al., 2019). Indeed, CART(42–89)_9–28_, a shorter variant of the CART protein, led to GPR68‐dependent mass redistribution responses, suggested to reflect numerous intracellular events, including protein trafficking and receptor internalisation, with both sub‐ and low‐micromolar potencies (Foster et al., 2019). CART(42–89)_9–28_, along with two other peptides (osteocrin_33–55_ and corticotropin), acted as positive allosteric modulators of GPR68 (Hauser et al., 2020). However, this research remains limited, and it is unclear whether CART peptides are able to stimulate GPR68 in the brain (Funayama et al., 2023). Of note, the primary signalling pathways for GPR68 appear to be G_s_ and G_q_ (Mogi et al., 2005), whereas CART is thought to act via G_i/o_‐coupled signal transduction (Lakatos et al., 2005; Somalwar et al., 2018), suggesting that GPR68 is unlikely to be a cognate receptor for CART.

Another oGPCR, GPR160, has recently been posited as a cognate receptor for CART, driven by observations that either a CART or GPR160 antibody were able to attenuate CART_55–102_ induced nociceptive responses in mice (Yosten et al., 2020). Additionally, CART_55–102_ stimulated *cfos* mRNA (a marker of neuronal activation) expression in KATOIII cells with endogenous expression of GPR160, and exogenous CART_55–102_ co‐immunoprecipitated with GPR160 antibody in KATOIII cell lysates (Yosten et al., 2020). Furthermore, CART_55–102_ stimulated ERK phosphorylation in PC12 cells, the only known cell line with specific binding of CART (Lin et al., 2011), which was attenuated via a GPR160 mRNA‐targeted small interfering RNA (Yosten et al., 2020). Subsequent work found that injection of GPR160 antibody, prior to CART peptide CART_55–102_, into the fourth ventricle prevented exogenous CART peptide‐induced reductions in food and water consumption in rats (Haddock et al., 2021). Although these studies provided promising evidence of GPR160 being a putative receptor of CART, they did not assess, or report, the specific binding and/or affinity of CART peptide to GPR160 (Haddock et al., 2021; Yosten et al., 2020). Importantly, a recent study found that the GPR160 antibody did not displace binding of either CART_55–102_ or CART_62–102_, nor did it compete with the specific binding site of the CART peptide in PC12 cells (Freitas‐Lima et al., 2023). Additionally, no GPR160 mRNA or protein was found in PC12 cells, suggesting that CART binding in PC12 cells occurs via a different receptor present in this cell line (Freitas‐Lima et al., 2023). Furthermore, saturation and competition binding assays in a THP1 cell line with high endogenous GPR160 expression revealed no specific binding, or competition, with CART peptide radioligands, strongly suggesting the lack of a CART receptor (Freitas‐Lima et al., 2023). Thus, the identity of a cognate CART receptor remains elusive and further work is needed. Without these developments, the full potential of targeting the CART system as a treatment for many neuropsychiatric disorders, including AUD, remains stalled.

## 3 ORPHANED GPCRs

### 3.1 GPR6

GPR6 is a constitutively active Class C GPCR that couples to a stimulatory G‐protein (G_S_) leading to increased cAMP levels at a similar amplitude of fully activated GPCRs (Tanaka et al., 2007; Uhlenbrock et al., 2002) and enhanced neurite outgrowth in vitro (Tanaka et al., 2007). *GPR6* mRNA is predominantly expressed in neurons in the brain, particularly in the striatum (caudate, putamen, nucleus accumbens, and olfactory tubercle) and to a lesser extent the frontal cortex, retrosplenial cortex, hippocampus, amygdala, and hypothalamus across species (Heiber et al., 1995; Marchese et al., 1994; Song et al., 1994). Within the striatum, GPR6 is localised on dopamine D_2_ receptor‐expressing striatopallidal medium spiny neurons (MSNs) (Heiman et al., 2008; Lobo et al., 2007). Controversy about the endogenous ligand for GPR6 still persists. Initial studies showed agonism of GPR6 by sphingosine‐1‐phosphate (S1P), but these results were not replicated (Ignatov et al., 2003; Yin et al., 2009), leaving GPR6 as an orphan receptor (Alexander et al., 2017).

GPR6 expression is most dense within the striatum, a brain region important for reward behaviours, decision making, and motor control (Lobo et al., 2007). Two intermingled but distinct populations of MSNs, differing in dopamine receptor subtype expression, control behavioural output from the striatum. Dopamine D_1_ receptor‐expressing MSNs in the dorsal striatum project into and inhibit the substantia nigra pars reticulata (SNr; direct, or striatonigral pathway), releasing inhibition of thalamic activity and therefore prompting motor output. In contrast, D_2_ receptor‐expressing MSNs project to and inhibit the external globus pallidus (indirect or striatopallidal pathway), disinhibiting the subthalamic nucleus and exciting the downstream SNr, which ultimately inhibits the thalamus and suppresses motor output (Kreitzer & Malenka, 2008). These pathways are postulated to antagonise each other to allow a balanced striatal output (Albin et al., 1989).

Dopamine is released in the dorsal striatum by neurons located in the substantia nigra pars compacta (SNpc) and acts upon D_1_ dopamine receptors (which depolarise the cell in response to dopamine) and D_2_ dopamine receptors (which hyperpolarise the cell in response to dopamine). Drugs of abuse acutely increase dopamine release in the striatum and thus have the dual effect of exciting the direct pathway, while simultaneously inhibiting the indirect pathway (Kreitzer & Malenka, 2008). GPR6 is specifically expressed in striatopallidal, D_2_ receptor‐positive neurons (Heiman et al., 2008; Lobo et al., 2007), and deletion of these receptors leads to adaptive changes in both striatopallidal‐specific genes (*DRD2* and *ADORA2A)* and a striatonigral‐specific gene (Lobo et al., 2007). Further, GPR6‐deficient mice have increased dopamine and metabolite levels in the striatum (Oeckl et al., 2014) and enhanced instrumental responding for a sucrose reward, without altered motor coordination (Lobo et al., 2007).

Genome‐wide RNA sequencing has recently shown GPR6 is down‐regulated in the dorsal striatum of individuals with AUD (Walker et al., 2020). In the prefrontal cortex (PFC) however, sequencing revealed *Gpr6* was up‐regulated in both food and cocaine “addicted” compared with “non‐addicted” mice, with an addiction criteria‐index based upon operant self‐administration behaviour (cocaine intake, total lever presses, active and inactive lever discrimination, consummatory regulation) used to determine designation of a cocaine addiction‐like phenotype (Navandar et al., 2021). However, the exact role of GPR6 in driving alcohol and substance use is not known; whether dysregulation of GPR6 signalling is causal or a consequence of substance use is not established and whether targeting GPR6 may have potential to reduce alcohol and substance use requires elucidation.

Recently, several small molecule inverse agonists have been identified and developed to interact with GPR6. Phylogenetically, GPR6 is closely related to the cannabinoid receptors, and cannabidiol (CBD), several synthetic cannabinoids, and endocannabinoid‐like N‐acylamides act as inverse agonists at GPR6 (Laun et al., 2019; Laun & Song, 2017; Shrader & Song, 2020). Preclinical studies in rats and mice have shown that cannabidiol may be effective in decreasing opioid, psychostimulant, nicotine, and alcohol use (Nona et al., 2019; Prud'homme et al., 2015) and 8 clinical trials for cannabidiol in AUD are in progress (Table 1). However, the mechanisms that cannabidiol acts through are widespread, and whether any actions are mediated via GPR6 would require further examination. A novel compound, CVN424, has recently been developed and shown to be a potent, orally active, and brain‐penetrant selective inverse agonist for GPR6, which is effective in reducing Parkinson's‐like symptoms in a rodent model (Brice et al., 2021). This compound has successfully undergone Phase I safety trials and is currently in Phase II trials (Margolin et al., 2022), highlighting a potential future opportunity of repurposing for other indications. However, given the opposing regulation of GPR6 in the striatum and PFC in response to drugs of abuse, whether inverse agonism would further exacerbate symptoms is a possibility that may limit development in this regard.

### 3.2 GPR26

GPR26, first cloned in 2000, is a Class A (Rhodopsin) GPCR that couples to G_s_ and promotes constitutive activation of the adenylyl cyclase pathway (Jones et al., 2007; Lee et al., 2001). GPR26 is brain specific, with enriched expression observed within the cortex, amygdala, hippocampus, hypothalamus, thalamus, and midbrain ventral tegmental area (VTA)‐SN (Ehrlich et al., 2018; Jones et al., 2007; Lee et al., 2001; Zhang et al., 2011). Early studies hypothesised GPR26 to be activated by nucleoside diphosphates and triphosphates based on its sequence homology with purinergic P2Y receptors; however, this was not confirmed (Lee et al., 2001) and GPR26 remains orphaned.

GPR26 mRNA and protein are found in several brain regions critical in regulating reward processing including the amygdala and midbrain VTA‐SN (Jones et al., 2007). While little research has directly explored the role of GPR26 in alcohol or substance use disorders, using a novel *Gpr26* KO mouse line, Zhang et al. (2011) showed male *Gpr26* KO mice consumed more alcohol than WT controls in a two‐bottle free‐choice paradigm. However, this was only observed at a low concentration of alcohol (7% v/v), and at higher concentrations (9% and 12% v/v), alcohol‐drinking behaviours were similar to WT (Zhang et al., 2011). This same study linked GPR26 to anxiety and depression, two disorders that are often co‐morbid with alcohol and other substance use disorders (Boden & Fergusson, 2011; Sinha, 2012; Walker, 2021). *Gpr26*‐deficient mice showed heightened levels of anxiety‐like behaviour in the elevated plus maze and open field test and increased depression‐like behaviours in the Porsolt swim and tail suspension tests (Zhang et al., 2011). However, the links between aberrant anxiety‐ and depressive‐like behaviour and alcohol consumption were not explored. *Gpr26* KO mice also displayed reduced CREB phosphorylation in the CeA compared with WT counterparts (Zhang et al., 2011), a process that has been linked to heightened anxiety and alcohol consumption (Pandey et al., 2005). However, little further exploration has arisen from these studies, and no novel targeting methods have been developed to further explore the links between GPR26, anxiety, depression, and alcohol intake.

### 3.3 GPR88

GPR88 is a brain‐specific G_i/o_‐coupled GPCR that is densely expressed in GABAergic MSNs in the striatum (Massart et al., 2009; Mizushima et al., 2000). Lower expression of GPR88 has also been reported in the olfactory tubercle, cortex, thalamus, and inferior olivary nucleus of *Gpr88‐*Cre mice and Sprague–Dawley rats (Ghate et al., 2007; Quintana et al., 2012; Van Waes et al., 2011). Within the striatum, GPR88 is expressed in both dopamine D_1_‐ and D_2_‐receptor expressing MSNs, and it is primarily localised in dendritic spines containing vesicular glutamate transporter 1 (VGLUT1), but not VGLUT2 or tyrosine hydroxylase (Massart et al., 2009; Quintana et al., 2012). The involvement of GPR88 in distinct behaviours appears to be cell‐type specific. GPR88 in D_2_ receptor‐MSNs was implicated in shaping social and defensive behaviours and in sustaining inhibition of basal ganglia coordination of locomotion and motor coordination. In contrast, GPR88 activation in D_1_ receptor‐MSNs in the striatum promotes novelty habituation and motor learning (Meirsman et al., 2019).

The striatum is also a critical brain region involved with decision‐making, reward‐seeking, and addiction (Kalivas & Volkow, 2005; Massart et al., 2009). Depletion of GPR88 increases MSNs excitability via glutamatergic and autoreceptor regulator of G protein signalling 4 (RGS4)‐dependent GABA signalling (Quintana et al., 2012). With the development of *G* *pr88* KO mice, Meirsman and collaborators showed a role for GPR88 expressed in adenosine A_2A_ receptor /D_2_ receptor‐expressing striatal neurons in increasing trait anxiety‐like behaviours without affecting other associated behaviours such as conflict anxiety and fear (Meirsman, Le Merrer, et al., 2016; Meirsman, Robe, et al., 2016). Given the well‐defined role for the striatum in AUD and the emerging research on striatal GPR88 contribution to behaviours such as poor motor coordination, impaired cue‐based learning, and hyperactivity in both rodents (Logue et al., 2009; Maroteaux et al., 2018; Quintana et al., 2012) and humans (Alkufri et al., 2016), it comes with no surprise that GPR88 activity in this brain region might be important for the development and maintenance of addiction‐like behaviours associated with alcohol use.

*Gpr88* KO mice show increased voluntary alcohol intake and motivation to acquire alcohol, but not other palatable rewards (Ben Hamida et al., 2018). Additionally, alcohol‐induced dopamine release in the nucleus accumbens was reduced, suggesting decreased reward‐driven alcohol consumption and/or consumption of alcohol driven by habitual behaviour in *Gpr88* KO mice (Ben Hamida et al., 2018). In addition, previous research has shown that *Gpr88* KO mice have lower basal extracellular dopamine in the striatum, but amphetamine‐induced dopamine release was normal, suggesting a role for this receptor in dopamine signalling regulation in the striatum (Logue et al., 2009). Altogether, these data suggest that targeting this receptor could have therapeutic effect to treat AUD.

Preclinical studies targeting GPR88 have shown efficacy of the agonist RTI‐13951‐33, derived from the 2‐PCCA ((1*R*, 2*R*)‐2‐pyridin‐2‐yl‐cyclopropane carboxylic acid ((2*S*, 3*S*)‐2‐amino‐3‐methyl‐pentyl)‐(4′‐propylbiphenyl‐4‐yl)‐amide) scaffold, in reducing alcohol self‐administration and intake in rats, in a dose‐dependent manner, without impairing locomotion (Jin et al., 2018). This agonist is potent, brain penetrant and selective to the GPR88 receptor. In contrast, a study found that in *Gpr88* KO mice, intraperitoneal injection of this agonist decreased locomotor activity, as well as reducing voluntary alcohol drinking, suggesting a GPR88 independent mechanism of action (Ben Hamida et al., 2022). Similar results have been previously reported where the GPR88 agonist 2‐PCCA dose‐dependently decreased locomotor activity in rats (Li et al., 2013). These distinct behavioural outcomes may be attributed to the differences between administering a GPR88 receptor agonist, in naïve animals, compared with transgenic *Gpr88* KO mice. Indeed, genetic compensatory mechanisms have been shown across vertebrate and invertebrate models (El‐Brolosy & Stainier, 2017) but not specifically examined in *Gpr88* KO mice. Nonetheless, further pharmacological characterisation of RTI‐13951‐33 is needed to elucidate its mechanism and therapeutic potential. These data do however support a theoretical rationale for further assessment of GPR88 targeted treatments for AUD.

### 3.4 GPR139

GPR139 is a Class A peptide receptor first discovered in 2002 and discretely expressed within the human and rodent brain (Takeda et al., 2002). The highest expression of GPR139 mRNA is observed in the dorsal medial habenula, with lesser expression in the VTA, dorsal striatum, nucleus accumbens, lateral septum, hypothalamus, and medial mammillary nucleus (Liu et al., 2015; Matsuo et al., 2005; Susens et al., 2006; Wang et al., 2015). Previous research, using a GPR139 plasmid transfected into CHO‐K1 cells, suggested that GPR139 signalling was dependent on receptor coupling to an inhibitory G‐protein and phospholipase C enzyme action, as well as dimerisation for proper function (Susens et al., 2006). Further, and similar to GPR88 signalling, GPR139 interacts with both μ‐opioid and dopamine D_2_ receptors in the brain (Rabiner et al., 2022), also suggesting a role for GPR139 in complex CNS processes. Indeed, research using *Gpr139* KO mice showed that loss of this receptor led to a series of behavioural abnormalities including motor and cognitive deficits and anxiolytic traits, along with evidence of dysfunctional μ‐opioid and dopamine D_2_ receptor signalling underlying these abnormalities (Dao et al., 2022). Further research into the interaction between GPR139 and the opioid system found that this receptor activation led to anti‐opioid activity in *Caenorhabditis elegans* (Wang, Stoveken, et al., 2019). This was conserved in rodents, where deletion of GPR139 in mice potentiated opioid‐induced inhibition of neuronal firing (Wang, Stoveken, et al., 2019). These data were further corroborated by electrophysiological data from *Gpr139* KO mice medial habenula neurons, where GPR139 receptor signalling was suggested to prevent μ‐opioid receptor‐mediated neuronal inhibition via G_q/11_ coupling (Stoveken et al., 2020). Further, *Gpr139* and *Drd2* mRNA are co‐expressed in several other brain regions implicated in AUD, including the caudate putamen, lateral septum, lateral habenula, VTA and arcuate nucleus of rats, mice, and humans (Wang, Lee, et al., 2019), but their interactions have not been explored in regard to alcohol or substance use in these regions.

GPR139 binding may not be restricted to one endogenous ligand, as recent research has implicated adrenocorticotropic hormone and melanocyte‐stimulating hormone α and β subunits as agonists at this receptor (Nohr et al., 2017). In addition, physiological concentrations of L‐tryptophan and L‐phenylalanine can activate this receptor (Liu et al., 2015; Shoblock et al., 2019). Nonetheless, GPR139 remains an orphan receptor to date.

Within the medial habenula, GPR139‐positive neurons, which also co‐express μ‐opioid receptors, send indirect downstream projections to the interpeduncular nucleus, where GPR139 receptors are also present (Boulos et al., 2017; Liu et al., 2015). These two brain regions form an important relay centre between limbic systems and midbrain and hindbrain, having roles in addiction, anxiety, and emotional processing (Batalla et al., 2017; Bianco & Wilson, 2009; Ehrlich et al., 2018; Fowler & Kenny, 2012). Indeed, signalling between the medial habenula and the interpeduncular nucleus has been associated with alcohol, nicotine, opiate, and stimulant dependence (McLaughlin et al., 2017). Nonetheless, research in alcohol‐dependent rats suggested that compulsive‐like drinking and decreased withdrawal‐induced hyperalgesia was not mediated by the medial habenula‐interpeduncular nucleus circuit, but by the activity of GPR139 itself within the medial habenula (Kononoff et al., 2018).

Organic ligands that target GPR139 and present drug‐like properties have been developed in recent years. JNJ‐63533054 is a small molecule agonist that is orally bioavailable and can cross the blood–brain barrier (Shoblock et al., 2019). A preclinical study reported that JNJ‐63533054 reduced escalation of alcohol self‐administration in alcohol‐dependent male rats, in a dose‐dependent manner, when administered systemically (Kononoff et al., 2018). Similarly, Wang et al. (2020) showed that JNJ‐63533054 suppressed morphine intake in morphine‐dependent mice (Wang et al., 2020). These data demonstrate the role of GPR139 in negatively regulating opioid and alcohol intake and highlight the potential of pharmacologically targeting this receptor. Analogous to JNJ‐63533054, TAK‐041 (also known as NBI‐1065846) is a novel potent GPR139 agonist with a favourable pharmacokinetic profile (Reichard et al., 2021). This compound increased sociability in mice with social interaction deficits (Reichard et al., 2021) and increased effort to obtain food in mice that were moderately food deprived (Munster et al., 2022) via GPR139 signalling. Indeed, a potential role for GPR139 in motivational processes has been previously described. Munster and collaborators reported that *Gpr139* KO mice display profound impairment in gustatory reward acquisition in an operant task that was rescued by TAK‐041 delivered orally (Munster et al., 2022). TAK‐041 was safe and well tolerated in healthy volunteers and patients with schizophrenia following Phase I clinical trials (Yin et al., 2022) and has undergone Phase II clinical trials investigating its effects on motivational anhedonia in patients with stable schizophrenia (trial ID NCT03319953) and to determine the effects of this ligand on amphetamine‐induced dopamine release in the brain (ID NCT02959892). Currently, a Phase II trial (NCT05165394) is assessing the efficacy of TAK‐041 in improving symptoms of anhedonia in patients with major depressive disorder, highlighting the pharmacological potential of this molecule.

### 3.5 GPR158

GPR158 is a Class C oGPCR, discovered in 2005 through genome assembly and GPCR gene predictions (Bjarnadottir et al., 2005). It is one of the most abundant oGPCRs in the brain, expressed throughout the CNS, including the prefrontal cortex, hippocampus, striatum, and cortex (Chang et al., 2023). Interestingly, GPR158 does not signal through traditional Class C GPCR mechanisms; instead, it localises regulator of G protein signalling 7 (RGS7), G_β_5 and allosterically promotes GTPase activity of Gα_i/o_ (Hajj et al., 2019). This ultimately reduces the activity of adenylate cyclase and influences downstream signalling pathways and subsequent depressive‐like behaviour (Hajj et al., 2019; Orlandi et al., 2015; Song et al., 2019). Previously, osteocalcin and heparan sulphate proteoglycans were proposed ligands for GPR158 (Khrimian et al., 2017). Further, a recent study suggests that glycine acts as an endogenous ligand at GPR158, inhibiting formation of the RGS7–Gβ5 complex and cAMP to regulate neuronal excitability, suggesting that this GPCR may have been adopted as a metabotropic glycine receptor (Laboute et al., 2023); however, further validation is required. The crystal structure of GPR158 both alone and bound to RSG7 and G_β_5 have recently been solved by two groups (Jeong et al., 2021; Patil et al., 2022), which may accelerate drug discovery and development of GPR158 ligands.

GPR158 has been implicated in depressive disorders. In humans, GPR158 mRNA is up‐regulated in the PFC of individuals with major depressive disorder, which is conserved in rodent models of stress‐induced depressive behaviour and can be rescued through genetic manipulation of GPR158 (Sutton et al., 2018). Interestingly, our recent genome‐wide RNAseq analysis showed reduced *GPR158* expression within the striatum of individuals with AUD (Walker et al., 2020), suggesting region‐specific actions and regulation of GPR158 may occur. Given the link between stress, depression and alcohol use (Boden & Fergusson, 2011; Gilpin et al., 2015; Sinha, 2012; Walker, 2021), GPR158 may also be effective for the treatment of alcohol and substance use disorders. GPR158 has recently been shown to mediate sensitivity to the sedative effects of ethanol (Wei et al., 2023). Thus, *Gpr158* null mice had a deficit in recovery from a sedative dose of alcohol (3.5 mg·kg^−1^) without altering alcohol metabolism or basal differences in locomotor activity, or basal anxiety‐like behaviour (Wei et al., 2023). Further, using a *Gpr158*‐floxed mouse, they showed that this effect was, in part, driven by GPR158 expression on both glutamatergic and GABAergic populations in the brain (Wei et al., 2023). However, studies to date have not reported the effect of either knockout mice, or selective drug targets, in reducing alcohol or drug consumption, self‐administration, or relapse.

## 4 CONCLUDING REMARKS

AUD remains a major socioeconomic burden, and treatment novel options remain elusive. Several orphaned neuropeptides and oGPCRs are expressed throughout key reward circuitry and may provide novel targets and treatments for AUD; however, limitations remain. Despite considerable efforts in the field of GPCR deorphanisation, more than 100 receptors remain orphaned, and these receptors are also disproportionally understudied (Alexander, Christopoulos et al., 2021; Roth & Kroeze, 2015). The challenges with orphaned peptides and receptors persist, including technical (limited availability of sensitive screening assays) or ability to test appropriate ligands and biological (a possible lack of endogenous ligand or receptor). In some instances, orphan peptides and receptors may have become evolutionarily redundant, or receptors may only signal through ligand‐independent mechanisms (e.g. constitutive activity or dimerisation) (Fricker & Devi, 2018; Tao & Conn, 2014). With the development of integrated computational, structural, functional, and experimental approaches, elucidating orphan peptide and orphan receptor interactions through screening putative receptors/ligands in silico are promising areas of future research (Foster et al., 2019). Further, advancements in cryo‐EM have enhanced exploration into membrane‐bound GPCRs in both active and inactive state conformations. In addition, artificial intelligence approaches, such as machine learning, are increasing efficiency in drug design and development (Casadó & Casadó‐Anguera, 2023), including several GPCR targeting compounds that have progressed to Phase I/II clinical trials, such as EXS21546, a selective A_2A_ receptor antagonist for renal cell carcinoma—clinicaltrials.gov ID: NCT04727138 & NCT05920408). We have outlined several targets that should be further explored as potential treatment options for AUD. However, limitations in small molecule compounds and mechanistic understanding are currently halting development. Leveraging emerging technologies will both enhance our fundamental understanding of orphaned peptides and receptors and provide a more rapid and precise method to identify pharmacotherapies to provide more treatment options for AUD.

### 4.1 Nomenclature of targets and ligands

Key protein targets and ligands in this article are hyperlinked to corresponding entries in https://www.guidetopharmacology.org/ and are permanently archived in the Concise Guide to PHARMACOLOGY 2021/2022 (Alexander, Christopoulos et al., 2021; Alexander, Fabbro et al., 2021; Alexander, Kelly, Mathie, Peters, Veale, Armstrong, Faccenda, Harding, Pawson, Southan, Buneman et al., 2021; Alexander, Kelly, Mathie, Peters, Veale, Armstrong, Faccenda, Harding, Pawson, Southan, Davies et al., 2021).

## AUTHOR CONTRIBUTIONS

**Roberta Goncalves Anversa:** Conceptualization (equal); project administration (equal); writing—original draft (equal); writing—review and editing (equal). **Xavier J. Maddern:** Conceptualization (equal); writing—original draft (equal); writing—review and editing (equal). **Andrew J. Lawrence:** Writing—review and editing (equal). **Leigh C. Walker:** Conceptualization (lead); writing—original draft (lead); writing—review and editing (equal).

## CONFLICT OF INTEREST STATEMENT

All authors report no conflicts of interest.

## Data Availability

No new data has been generated.
